# Proteolytic activity and degradation of bovine versus human dentin matrices

**DOI:** 10.1590/1678-7757-2021-0290

**Published:** 2021-12-01

**Authors:** Cristiane Mayumi Inagati, Débora Lopes Salles Scheffel, Giovana Anovazzi, Juliana Rosa Luiz Alonso, Marcelly Tupan Christoffoli, David Henry Pashley, Carlos Alberto De Souza Costa, Josimeri Hebling

**Affiliations:** 1 Universidade Estadual Paulista Faculdade de Odontologia de Araraquara Departamento de Materiais Dentários e Prótese Araraquara São Paulo Brasil Universidade Estadual Paulista (UNESP), Faculdade de Odontologia de Araraquara, Departamento de Materiais Dentários e Prótese, Araraquara, São Paulo, Brasil.; 2 Universidade Estadual de Maringá Departamento de Odontologia Maringá Paraná Brasil Universidade Estadual de Maringá (UEM), Departamento de Odontologia, Maringá, Paraná, Brasil; 3 Universidade Estadual Paulista Faculdade de Odontologia de Araraquara Departamento de Morfologia e Clínica Infantil Araraquara São Paulo Brasil Universidade Estadual Paulista (UNESP), Faculdade de Odontologia de Araraquara, Departamento de Morfologia e Clínica Infantil, Araraquara, São Paulo, Brasil.; 4 Augusta University Department of Oral Biology The Dental College of Georgia Augusta Georgia United States Augusta University, Department of Oral Biology, The Dental College of Georgia, Augusta, Georgia, United States.; 5 Universidade Estadual Paulista Faculdade de Odontologia de Araraquara Departamento de Fisiologia e Patologia Araraquara São Paulo Brasil Universidade Estadual Paulista (UNESP), Faculdade de Odontologia de Araraquara, Departamento de Fisiologia e Patologia, Araraquara, São Paulo, Brasil.

**Keywords:** Dentin, Animals, Collagen, Matrix metalloproteinases, Hydroxyproline

## Abstract

Non-human teeth have been commonly used in research as replacements for human teeth, and potential dissimilarities between the dental tissues should be considered when interpreting the outcomes.

**Objective:**

To compare the proteolytic activity and degradation rate of bovine and human dentin matrices.

**Methodology:**

Dentin beam specimens were obtained from human molars (n=30) and bovine incisors (n=30). The beams were weighed hydrated and after complete dehydration to obtain the mineralized wet and dry masses. Then, the beams were demineralized in 10 wt% phosphoric acid. Next, 15 beams from each substrate were randomly selected and again dehydrated and weighed to obtain the initial demineralized dry mass (DM). Then, the beams were stored in saliva-like buffer solution (SLBS) for 7, 14 and 21 days. SLBS was used to evaluate hydroxyproline (HYP) release after each storage period. The remaining beams of each substrate (n=15) were tested for initial MMP activity using a colorimetric assay and then also stored in SLBS. DM and MMP activity were reassessed after 7, 14 and 21 days of incubation. The data were subjected to two-way ANOVA tests with repeated measures complemented by Bonferroni’s tests. Unpaired two-tailed t-tests were also used (p<0.05).

**Results:**

Similar water and inorganic fractions were found in human and bovine dentin, while human dentin had a higher protein content. The most intense proteolytic activity and matrix deterioration occurred short after dentin was demineralized. Both substrates exhibited a sharp reduction in MMP activity after seven days of incubation. Although human dentin had higher MMP activity levels, greater HYP release and DM loss after seven days than bovine dentin, after 14 and 21 days, the outcomes were not statistically different.

**Conclusion:**

Bovine dentin is a suitable substrate for long-term studies involving the degradation of dentin matrices.

## Introduction

Human teeth are the ultimate clinically-relevant substrate for dental research.^1^ However, many studies have used non-human alternative substrates,^1^ since human teeth may be difficult to obtain in large quantities from controlled sources and at particular ages.^2,3^ Moreover, human teeth provide relatively restricted enamel and dentin areas, have infection hazards and ethical concerns.^1-3^Among non-human tooth substitutes, bovine incisors have been the most used substrate.^1^ Bovine incisors have been used for dental caries^4^, dental erosion/abrasion,^5^ microleakage,^6^ dental bleaching,^7^ bond strength,^8^ collagen biomodification,^9^ protease inhibition, and remineralization studies.^10^ Bovine incisors offer wide enamel, coronal and root dentin areas, besides reducing variables hard to control such as age, diet, and other environmental conditions. Furthermore, bovine incisors can be easily obtained in large number and good quality.^1^

Although several similarities led to the conclusion that both bovine incisor enamel and dentin are reliable replacement substrates for human tissues when used in bonding studies,^8^ to the best our knowledge, no studies have compared the proteolytic activity exerted by dentin matrix metalloproteinases (MMPs) and cysteine cathepsins (CTs) of bovine incisors and human dentin. Non-collagenous proteins (NCPs) comprise 10% of the human dentin protein content.^11^ Among them, MMPs and CTs are endopeptidases responsible for breaking down proteins of the extracellular matrix (ECM), including type I collagen, which is the highest protein in dentin (90% of the protein content) and the main component of the hybrid layer along with the adhesive resin.

MMP-2 (gelatinase), MMP-8 (collagenase), MMP-9 (gelatinase), CT-B and CT-K have been widely reported as important contributors to resin-dentin bond degradation, since they hydrolyse exposed collagen fibrils within the hybrid layer.^12,13^ The almost complete disappearance of hybrid layers has been reported after only six months from the placement of composite resin restorations in the oral cavity, in contrast with the small and scarce areas of degradation seen in hybrid layers produced in the presence of a non-specific MMP and CCs inhibitor.^14^ The enzymatic degradation mediated by these dentin proteases results in an approximately 43% reduction in bond strength in less than four months.^15^

MMP-2 and -9 have been detected in both human and bovine dentin, with similar enzyme activity for MMP-9. However, human dentin showed 30% higher MMP-2 levels both for root and crown samples.^16^ The higher MMP-2 activity was associated with greater numbers of tubules in human dentin. Thus, the differences in the tubule structure and morphology of both substrates^17^ must be considered in such studies. Considering the lack of evidence regarding the proteolytic activity and matrix degradation of bovine dentin, this study aimed to compare the *in situ* activity of MMPs and the degradation rate of human versus bovine dentin matrices. The tested null hypotheses were that bovine and human main dentin composition were similar and that there was no difference in the proteolytic activity or the degradation rate between the two substrates up to 21 days of storage in saliva-like buffer solution.

## Methodology

### Experimental design

In total, 30 dentin specimens (beams) were cut from bovine incisors and the same number of specimens was cut from human molars. Dentin specimens were evaluated for (1) MMP activity, (2) dry mass change and (3) hydroxyproline (HYP) release in three different time points, 24 hours, 7 and 21 days, after storage in a saliva-like buffer solution. The final number of specimens used for each experimental protocol was estimated to obtain a minimum power of 80%, and, although the test suggested nine specimens, 15 specimens were used, considering possible losses. “Tooth type” and “storage period” were designed as the factors of the study, with two (bovine and human) and three levels (7, 14 and 21 days), respectively. Response variables were MMP activity, dry mass change and release of HYP. Data were considered independent when comparing type of tooth within each time point and repeated measures when comparing the time points within the same tooth type.

### Dentin specimen preparation

A total of 15 sound bovine incisors and 15 sound third human molars were used in this study. Human teeth were collected after approval by the Ethics Committee for Human Research (CAAE 55962515.6.0000.5416) from patients between 18-23 years of age. Bovine incisors were obtained from animals aged approximately 3 years in a slaughterhouse. After cleaning with deionized water and prophylaxis using a pumice slurry, all extracted teeth were immersed in phosphate buffered solution (PBS) containing 0.1% thymol and stored at 4°C for no longer than 30 days.Then, one 0.5 mm-thick dentin disc (n=15) was cut from the middle third of each human molar crown using a metallographic saw (IsoMet 1000, Buehler Ltd, Lake Bluff, IL, USA) equipped with a 0.3 mm-thick diamond blade (Diamond Wafering Blade, Buehler Ltd), and under constant water irrigation. Each disc was further sectioned to produce two 0.5 mm-thick, 1.0 mm-wide and 4.0 mm-long dentin beams (n=30). The buccal surface of bovine incisors was abraded in a rotary politrix (Beta 2; Buehler Ltd, Lake Bluff, IL, USA) using 320-grit carbide papers until a flat dentin surface was obtained. Then, the buccal and lingual surfaces of the bovine incisors were separated using the metallographic saw, and the buccal surfaces were used to obtain 30 specimens (0.5 x 1.0 × 4.0 mm). The dimensions of the beams were confirmed using a digital calliper with a 0.01 mm resolution (Mytutoyo Sul Americana Ltda., São Paulo, SP, Brazil), and after they were used in the experimental protocols.

### Mineralized mass and collagen matrices preparation

A total of 15 beams from each substrate were randomly rinsed in deionized water and blot dried with absorbent paper. Each beam was weighed using a microanalytical precision balance with 0.1 µg readability (Microbalance XP6, Mettler-Toledo International, Inc., Columbus, OH, USA) to determine their mineralized wet mass (mg). Then, the beams were sealed in a desiccator with anhydrous calcium sulphate (Drierite, W.A. Hammond Drierite Company, Ltd., Xenia, OH, USA) for 72 h and the mineralized dry mass was measured. Next, all the beams from each substrate (n=30) were individually placed in 5 mL microtubes (Eppendorf; Hamburg, Germany) containing 3 mL of a 10 wt% phosphoric acid solution. The microtubes were positioned in a tube homogenizer at 12 rpm (Phoenix AP22, Luferco; SP, Brazil) and demineralized for 18 h at room temperature (25°C). Then, the demineralized beams were rinsed with deionized water for 2 h at 4°C.^18^ The same beams (n=15) which had the mineralized wet and dry masses measured were used to assess collagen degradation, while the remaining beams were tested for their MMP activity.

### Dry mass loss

Loss of dry mass was used to assess dentin collagen degradation. The demineralized beams from human and bovine incisors (n=15) were placed in a 96-well culture plate and then sealed in containers with anhydrous calcium sulphate for 72 h. The initial demineralized dry mass of each beam was measured.^19^ Then, the beams were individually stored in 300 μL of a saliva-like buffer solution (5 mM HEPES; 2.5 mM CaCl_2_.2H_2_O; 0.02 mM ZnCl_2_; pH 7.4^19^) at 37°C for 7, 14 and 21 days. The dry mass was re-measured after each incubation period, and the storage medium was frozen at -80°C for later HYP assay (n=15).

### Determination of dentin organic, inorganic and water fractions

The masses (mg) of mineralized, hydrated specimens corresponded to the whole mass of the specimen (m_esp_). Then, the mass of mineralized and dehydrated specimens was subtracted to calculate the water fraction (wf). The mass of demineralized and dehydrated specimens represented the organic, protein fraction (pf) of dentin. Finally, the following formula was used to calculate the mineral fraction (mf): mf = m_esp_-wf-pf, and all the values were transformed in percentage, considering the m_esp_ as 100%.

### Hydroxyproline (HYP) assay

Hydroxyproline (HYP) was used as a marker for dentin collagen degradation. The frozen saliva-like buffer samples (300 μL) were thawed at room temperature and transferred to a 96-well culture plate that was kept in a desiccator until solution evaporation. The remaining content of each well was subsequently resuspended in 70 μL of ultrapure water and transferred to 2 mL Eppendorf tubes. HYP release was quantified after solubilized collagen was hydrolysed using a Hydroxyproline Assay Kit (catalogue no. MAK008, Sigma-Aldrich-St. Louis, Missouri, USA). Then, 70 μL of 37% hydrochloric acid was added into each tube and the tubes were sealed. The tubes were kept in a dry bath (Kasvi, São José dos Pinhais, PA, Brazil) for 3 h at 120 °C followed by the addition of 2.5 mg activated charcoal (catalogue no. 242276, Sigma-Aldrich, St. Louis, Missouri, USA). The tubes were centrifuged for two minutes at 13,000 g, and 40 μL aliquots of the supernatant from each tube were transferred to a 96-well culture plate in duplicate. Next, 100 μL of chloramine T in buffer was added to each compartment, and the plate was kept at room temperature for five minutes. Subsequently, one-hundred μL of a p-dimethylaminobenzaldehyde + isopropanol solution was added to each well, and the plate was incubated for 90 minutes at 60°C. Finally, the plate was read in a spectrophotometer (Synergy HT microplate reader, BioTek Instruments, Winooski, VT, USA) at 560 nm, and the HYP release values (μg/mg of dentin) were calculated using a standard curve.

### *In situ* MMP activity

First, MMP activity was measured using a Sensolyte Generic MMP colorimetric assay kit (catalogue no. 72095, AnaSpec, Inc., Fremont, CA, USA). Then, each demineralized dentin beam (n=15) was transferred to a 96-well culture plate containing 100 μL of a buffer solution (Component C) to determine the initial MMP activity. Next, 200 μL of the substrate (a mixture of 100 μL of Component A + 4.9 mL of Component C) was added to each well. The dentin specimens were removed from the Sensolyte solution after 60 minutes at room temperature, abundantly rinsed with deionized water, and inserted into microtubes (Eppendorf; Hamburg, Germany) containing 300 μL of the saliva-like buffer solution. The absorbance of each well was read at 412 nm against blanks using a spectrophotometer (Synergy HT microplate reader, BioTek Instruments, Winooski, VT, USA). The same protocol was conducted to assess MMP activity after 7, 14 and 21 days of storage.

### Statistical analysis

All the protocols were performed in duplicate and the data from the first runs (n=7) were used to estimate the sample size with G*Power. MMP activity was considered the main outcome, and the fixed parameters were alpha 0.05, power 0.8, two-tailed test and N2/N1=1. The sample size for each substrate indicated by the test was a minimum of nine. The final number of replicates was 15 for each group and protocol. Statistical analysis was performed with GraphPad Prism version 8 software program (GraphPad Software, La Jolla, CA, USA). MMP activity, dry mass loss and HYP release data had normal distributions and were evaluated using two-way ANOVA tests with one repeated measure (storage period), complemented by Bonferroni’s multiple comparison test. Water, organic and mineral fractions of human and bovine dentin were compared using unpaired two-tailed t-tests. All statistical inferences were based on a 5% significance level (p<0.05).

## Results

### Dry mass loss

**Figure 1:**
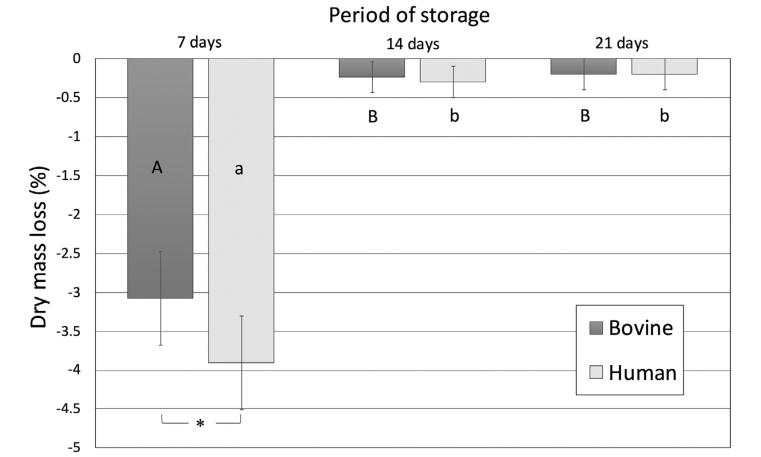
Dry mass loss (%) of human and bovine dentin matrices after up to 21 days of storage in saliva-like buffer solution. The mean initial mass (n=15) was considered 100% and was used to estimate the subsequent values. Letters allow comparisons within each substrate, while connectors indicate comparisons between the substrates for each period of storage. Different letters, as well as the presence of a connector and asterisk (*), indicate that the groups are significantly different (Bonferroni, p<0.05)

Figure 1 shows the dry mass loss percentages over time. Both bovine and human dentin lost mass over time. The dry mass loss was significant for both substrates for up to 14 days (p<0.0001). Human dentin presented a significantly higher mass loss after seven days (3.9±0.6% × 3.1±0.6%) (p<0.0001) in the saliva-like buffer solution. No difference was observed after 14 (0.24±0.2% × 0.3±0.2%) and 21 days (0.2±0.2 × 0.2±0.2) between the substrates (p=0.998).

### Bovine and human dentin organic, inorganic and water fractions

**Figure 2:**
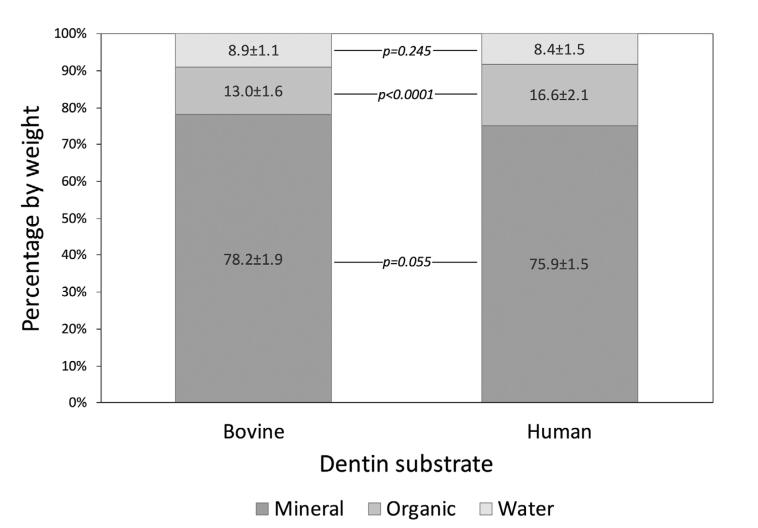
Percentage (by weight) of water, mineral and organic fractions of bovine and human dentin. p-values compare the same fraction of both substrates. Values <0.05 indicate statistically significant difference (t-test)

Figure 2 shows the weight percentage of each fraction of human and bovine dentin. Only the organic fraction was statistically different between the substrates (p<0.0001), with lower percentage of proteins found in bovine dentin.

### Hydroxyproline (HYP) release

**Figure 3:**
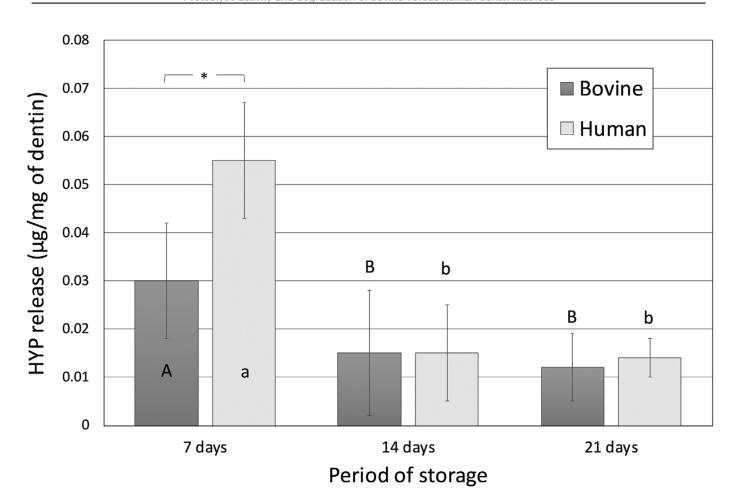
Amount of hydroxyproline (HYP) released from bovine and human dentin matrices up to 21 days of storage in saliva-like buffer solution. Letters allow comparisons within each substrate, while connectors indicate comparisons between the substrates for each period of storage. Different letters, as well as the presence of a connector and asterisk (*), indicate that the groups are significantly different (Bonferroni, p<0.05)

Figure 3 shows HYP release as μg/mg dentin. Human dentin matrices (0.055±0.012) released significantly more HYP after seven storage days than bovine dentin matrices (0.030±0.012) (p<0.0001). However, equivalent amounts were released after 14 days (0.015 ±0.010 - human × 0.015 ±0.013 - bovine) and 21 storage days (0.014 ±0.004 – human × 0.012±0.007 - bovine), with no significant difference between the two substrates (p>0.9999). The HYP release behavior was similar within each substrate, with higher amounts being released after seven days and subsequent smaller amounts after 14 and 21 days of storage.

### MMP activity

**Figure 4:**
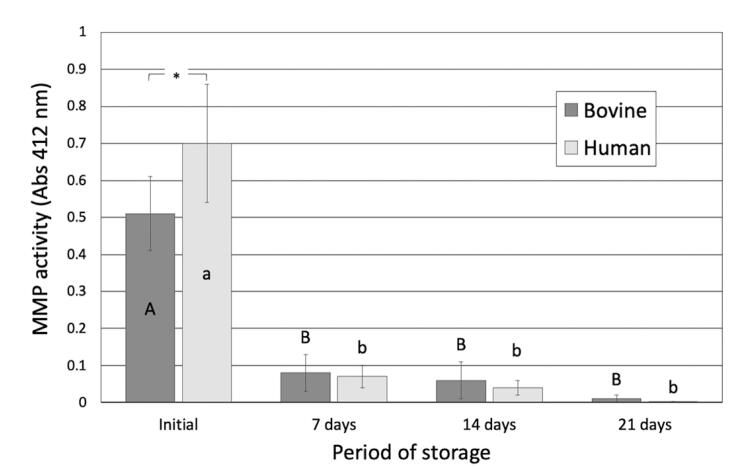
MMP activity expressed as absorbance detected in demineralized beam specimens obtained from bovine and human dentin after different periods of storage. Letters allow comparisons within each substrate, while connectors indicate comparisons between the substrates for each period of storage. Different letters, as well as the presence of a connector and asterisk (*), indicate that the groups are significantly different (Bonferroni, p<0.05)

The absorbance values of the colorimetric MMP assay were used to measure the MMP activity of each sample (Figure 4). MMP activity in the saliva-like buffer solution decreased significantly after each storage period for both bovine and human dentin matrices. It reduced drastically in the first seven days of incubation for both substrates, followed by a slow but significant reduction over time. Human dentin had significantly higher initial MMP activity (0.70±0.16) than bovine dentin (0.51±0.10) (p=0.0034).

## Discussion

To the best our knowledge, this is the first study to evaluate the proteolytic activity of bovine dentin over time compared to human dentin. Dentin is a highly mineralized complex tissue mainly composed of minerals, water, and proteins. Differences in the chemical, structural and anatomical characteristics of dentin may influence the results of studies using animal teeth instead of human teeth as a substrate and represent a variable that must be considered.^20^

Human dentin is composed of approximately 70% minerals, 10% water, and 20% proteins by weight^11^, which is slightly different from the composition found in this study. The mass of mineralized and demineralized wet and dry dentin matrix specimens was used to estimate the water percentage, as well as mineral and organic fractions in bovine and human dentin. No significant difference was observed between the human and bovine composition, except for the organic content, which was higher in human dentin. A lack of significant difference in the mineral content of human and bovine dentin has been previously reported.^21^ Both substrates contain similar Ca and P concentrations,^22^ radiodensity,^23^ and Knoop hardness.^24^ Human dentin also seems to present a slightly larger intertubular area and a better organized structure with lower number of defects compared to bovine dentin.^24^

The organic content of bovine dentin found in this study was significantly lower than human dentin, which was previously reported using FT-Raman spectroscopy.^25^ Therefore, the null hypothesis that bovine and human dentin have similar basic composition was rejected. Nevertheless, type I collagen is the major component of both human and bovine dentin matrices.^26^ Collagen serves as a scaffold to the three-dimensional structure of dentin and provides dentin with tensile strength, resistance, and cohesiveness.^27^ Moreover, collagen is extremely important in Adhesive Dentistry, since the collagen fibril network is infiltrated by adhesive monomers to provide micromechanical retention for adhesive restorations.^28^

The higher organic content of human dentin may explain its increased initial MMP activity compared to bovine dentin. A significant difference in proteolytic activity, mass loss and HYP release was seen only after seven days of storage. After 14 and 21 days, the proteolytic activity of both substrates was similar. Therefore, the null hypothesis that there was no difference in the proteolytic activity or the degradation rate between the two substrates up to 21 days of storage in saliva-like buffer solution was only partially accepted. The collagenolytic/gelatinolytic assay used in this study (Sensolyte) can measure the activity of MMP-1, -2, -3, -7, -8 -9, -10, -11 and -12. This assay has been used in multiple studies to identify the MMP activity in dentin beams, as well as the effectivity of MMP-inhibiting agents.^18,29-31^ A limitation of this method is that the Sensolyte substrate may not have reached the totality of MMPs in the inner portions of the specimens. The use of dentin powder could enable a better access to the enzymes. Several MMPs have been reported in human dentin.^13,32^ MMP-2 in human coronal dentin is more abundant^13^ in a 90–200 μm zone adjacent to predentin, as well as a 9–10-μm-wide zone adjacent to the dentinoenamel junction (DEJ). MMP-2 has also been found in human middle dentin in association with the dentin matrix.^33^ MMP-2 activity has been reported around 30% higher in the coronal and root human dentin when compared with bovine counterparts, while MMP-9 presented similar activity.^16^ This finding resembles the results of this study, which observed a 27% higher absorbance for the MMP activity of human dentin.

Both bovine and human dentin samples showed an abrupt reduction in MMP activity within the first seven storage days in the saliva-like buffer solution. A similar time-dependent decrease in proteolytic activity has previously been reported for human dentin.^34-35^ Matrix-bound MMPs could only cleave the substrate in the finite zone of their molecular mobility, which could lead to a restricted degradation of substrates and self-limiting collagen degeneration over time.^34-35^ The decrease in proteolytic activity over time may also explain the reduction in the dry mass loss rates and HYP release recorded after 14 and 21 storage days for both dentin substrates.

Hydroxyproline, an amino acid produced by the hydroxylation of proline, is found in large amounts in collagen (13-14%)^36^ and used as a biochemical marker of bone turnover and collagen type I degradation.^37^ HYP-containing peptide fragments released into the saliva-like buffer solution was used as a method in this study to evaluate the dentin matrix degradation rate over time. The highest HYP peptide release for both bovine and human dentin matrices was observed after seven storage days. This result is in accordance with the results observed for MMP activity and shows that collagen is intensely degraded within the first week of incubation. Human dentin released approximately 45% more HYP than bovine dentin, which may be explained by its higher organic mass (16.6% *vs.* 13.0%) associated with the elevated initial MMP activity.

Collagen cleavage results in releasing peptide fragments,^34-35,38^ culminating in mass loss over time. As expected, human dentin lost significantly more mass within the first seven days in the saliva-like buffer solution than bovine dentin due to the higher MMP activity and amount of HYP released. The average percentage of dry mass loss found in this study was lower than the values observed by Tezvergil-Mutluay, et al.^39^ (2011) for human dentin after seven storage days. This discrepancy may be explained by the differences in the composition of the saliva-like buffer solution used in each study. The saliva-like solution used by Tezvergil-Mutluay, et al.^39^ (2011) contained 0.05 mM ZnCl_2_,whereas this study used 0.02 mM ZnCl_2_. MMPs are calcium-zinc-dependent proteases, and these minerals play an important role in maintaining the enzyme tertiary structure and functional active sites.^19,40^ The catalytic domain of MMPs contains a Zn^2+^ion which is critical for both substrate binding and cleavage in such a way that changes in zinc levels may affect MMP activity.^41^

Both substrates presented a similar degradation pattern with an acute degradation phase within the first few days after demineralization, followed by a slower activity. However, the collagen degradation intensity was higher in human dentin. Thus, studies performed in bovine dentin, especially those which evaluate the ageing of resin-dentin bonds, should consider these findings when extrapolating their results to human teeth. Future studies should assess other biochemical markers for type I collagen degradation, such as ICTP cross-linked carboxyterminal telopeptide of type I collagen and CTX (C-terminal crosslinked telopeptide of type I collagen),^38^ and consider the role of other proteases such as cysteine cathepsins, on collagen breakdown in bovine dentin.

## Conclusion

Higher MMP activity and collagen breakdown were seen for human dentin in comparison to bovine dentin only in the first seven days, meaning that bovine dentin is a suitable substrate for long-term studies involving the degradation of dentin matrices, and that the results could be translated to human dentin.

## Footnotes

* This paper results from a dissertation that is publicly accessible at https://repositorio.unesp.br/bitstream/handle/11449/150930/inagati_cm_me_arafo_int.pdf?sequence=4&isAllowed=y
